# Identification of intraspecific cultivar *Melia azedarach* ‘Mizhi’ based on complete chloroplast genome data and leaf anatomy

**DOI:** 10.3389/fpls.2026.1783041

**Published:** 2026-03-12

**Authors:** Xiaoxuan Fan, Fengjiao Shen, Yaqiang Luan, Tao Dong, Chenxi Zhang, Fengwu Gou, Yitong Han, Wenhan Yao, Lu Wang, Xiaoman Wang, Zhengge Zhu, Yanlei Liu, Lin Li, Jiancheng Zhao, Zhibin Li, Shulei Jiang, Xiaoxia Bai, Shuo Shi

**Affiliations:** 1 College of Life Sciences, Hebei Normal University, Shijiazhuang, China; 2 Hebei Collaborative Innovation Center for Eco-Environment, Hebei Normal University, Shijiazhuang, China; 3 Hebei Normal University Museum, Hebei Normal University, Shijiazhuang, China; 4 School of Landscape and Ecological Engineering, Hebei University of Engineering, Handan, China; 5 Shijiazhuang Shenzhou Flower Institute Co. Ltd., Shijiazhuang, China; 6 Shijiazhuang Academy of Agriculture and Forestry Sciences, Shijiazhuang, China

**Keywords:** *Melia azedarach* ‘Mizhi’, complete chloroplast genome, cultivar identification, DNA barcoding, SNP, ITS

## Abstract

The *Melia azedarach* ‘Mizhi’ (cultivar ‘Mizhi’) is a recently developed cultivar within the genus *Melia*. It has an elegant tree form, dense foliage, and notable ornamental, ecological, and potential medicinal value, offering broad market prospects. As the number of cultivar *Melia* continues to increase, accurate identification of these cultivars becomes increasingly critical to ensure its proper utilization and successful large-scale promotion. This study aims to provide more evidence for cultivar identification, development and protection by analyzing shallow whole-genome sequencing data, leaf epidermal and cross-sectional structure. Finally, four complementary approaches: nuclear gene fragments, complete chloroplast genome data, leaf epidermal structure (include micromorphological and ultrastructural characteristic), and leaf cross-sectional structure, were used in this study. The results show that the *Melia azedarach* ‘Mizhi’ exhibits its uniqueness in all of the above aspects. Moreover, the SNP based on the chloroplast genome can directly reveal the differences between *Melia azedarach* ‘Mizhi’ and others. Overall, this study provides precise molecular markers for the identification and protection of *Melia azedarach* ‘Mizhi’, offering new technical tools and theoretical support for the sustainable development and utilization of *Melia* species. The standardized molecular markers and multi-scale phenotypic data generated in this study also provide high-quality source data for future AI-assisted and automated cultivar identification. This work lays a solid foundation for the development of objective, scalable, and data-driven identification systems in plant cultivar research.

## 1 Introduction

Although a wide diversity of varieties is currently available, the identification of cultivated varieties remains a major challenge. *Melia azedarach* L. (chinaberry, Persian lilac) is an important economic tree species widely distributed in tropical and subtropical regions and cultivated in some temperate areas. It has broad applications in medicine, landscaping, and industry. In terms of medicinal value, limonoids, triterpenoids, and other bioactive compounds present in *Melia* species exhibit significant pharmacological activities, such as anticancer, immunomodulatory, and insecticidal effects, and provide a rich material basis for new drug development (Chen et al., 2014; Feng et al., 2022; Kuniaki et al., 2020; Liu et al., 2025a). In horticultural applications, several species of the genus *Melia*, with their distinctive tree architecture, attractive flowers and fruits, rapid growth, and strong stress tolerance, serve as important plant materials for urban greening, garden beautification, and ecological landscape development (Feng et al., 2022). In addition, this species is widely used in the production of construction materials and furniture, as well as in the manufacture of agricultural tools, ships, vehicles, and musical instruments (Chen et al., 2014). At present, the development of *Melia* genetic resources has achieved notable progress. In addition to the traditionally cultivated *M. azedarach*, several distinctive cultivars have been bred, including ‘Mizhi’, ‘Zijini’ and ‘Ziyu’, and ‘Meiren’ (Bai et al., 2024; Fu et al., 2018; Jiang et al., 2022). However, as the commercial development of cultivars accelerates, problems such as cultivar misuse and germplasm confusion have become increasingly serious (Liu et al., 2024; Shen et al., 2023). The *M. azedarach* ‘Mizhi’ (cultivar ‘Mizhi’) is a recently developed cultivar within the genus *Melia*. The name ‘Mizhi’ means dense branches, reflecting the primary distinguishing characteristic of this cultivar. It has an elegant tree form, dense foliage, and notable ornamental, ecological, and potential medicinal value, offering broad market prospects. As the cultivar with the greatest application potential among the various *Melia* varieties, achieving precise identification of *M. azedarach* ‘Mizhi’ is urgently needed to ensure its accurate utilization and widespread promotion.

Compared with traditional macroscopic morphological traits such as leaf shape and inflorescence type, the micro-morphological characteristics of the leaf epidermis (including stomatal type, epidermal cell arrangement, and trichome distribution) and the anatomical features of transverse leaf sections (such as the number of palisade cell layers, the arrangement of vascular bundles in the veins, and the presence of secretory structures) provide greater stability and resolution for plant classification and identification. Because these microscopic traits are primarily determined by genetic factors and are less affected by environmental variation, they offer higher reliability for distinguishing closely related species or cultivated varieties (Albach et al., 2021). For example, the stomatal index--the ratio of stomatal density to the number of epidermal cells--and the ornamentation of the cuticle show significant interspecific variation and can serve as reliable taxonomic characters (Khan et al., 2022). In addition, leaf cross-sectional structures--such as the distinction between dorsiventral and isobilateral leaves and the distribution of sclerenchyma tissue--can reflect a plant’s ecological adaptations and provide complementary information to molecular phylogenetic data (Lagergren et al., 2023; Qiu et al., 2023).

Compared with traditional morphological traits, molecular marker techniques play an increasingly important role in plant cultivar identification and biodiversity conservation. In contrast to morphological traits, molecular markers are far less susceptible to developmental and environmental variation. They provide data that directly reflect the genotype, and their typically codominant nature and high polymorphism offer substantial informational content (Feng et al., 2020; Guo et al., 2023; Liu et al., 2025b). However, DNA barcoding, which is commonly used for species identification, is greatly limited in cultivar identification and protection due to its relatively low resolution (Azizi et al., 2021; Liu et al., 2025b; Shen et al., 2023; Shi, 2016). In view of this, researchers have begun to shift their focus toward the use of chloroplast whole-genome and even whole-genome “super barcodes” for identification purposes (Li et al., 2015). However, although “super barcodes” theoretically offer higher resolution and greater identification accuracy, their practical application in cultivar identification faces challenges such as high costs and the computational demands associated with large data volumes. This mismatch not only increases research expenses but may also lead to inefficiencies in data processing and analysis (Liu et al., 2021). Therefore, from a practical and rational perspective, the development of cultivar-specific molecular markers, such as insertion/deletion markers (InDel), simple sequence repeats (SSRs) markers, or single nucleotide polymorphism markers (SNPs), has become a more feasible approach. These specific markers not only allow for more precise cultivar identification but also effectively reduce research costs and improve identification efficiency (Lin et al., 2023). For *M. azedarach* ‘Mizhi’, the development of specific molecular markers can enable rapid and accurate identification of this cultivar, providing a scientific basis for its protection and application. At the same time, molecular marker techniques can be used to study the genetic diversity, population structure, and evolutionary relationships of *Melia* species, offering theoretical support for the conservation and rational utilization of *Melia* resources.

In this study, cultivars of *M. azedarach* were employed as model materials to systematically investigate cultivar identification through the integration of genomic and morphological evidence. By combining shallow whole-genome sequencing with detailed analyses of leaf epidermal and cross-sectional anatomical traits, we aimed to establish a robust framework for cultivar discrimination, development, and protection. Complete chloroplast genome sequences of *M. azedarach* cultivars were generated to comprehensively compare chloroplast genome organization and sequence variation among cultivars, and to critically assess their discriminatory power relative to conventional DNA barcoding markers. Integrating chloroplast genomic data with stable morphological characteristics enabled the development of cultivar-specific molecular markers for the accurate identification of the *M. azedarach* ‘Mizhi’. This study provides a theoretical and methodological foundation for the genetic improvement, precise identification, and broader utilization of *M. azedarach* cultivars.

## 2 Materials and methods

### 2.1 Sample collection and processing

Sampling also included common wild species and cultivated varieties, with a total of 49 *Melia* specimens analyzed comparatively. Fresh leaves of 49 samples of *M. azedarach* cultivars, were collected. The leaf surfaces were wiped with 75% ethanol to prevent contamination from fungi or other sources that could affect total DNA extraction. The leaves were then dried using silica gel for subsequent experiments. Voucher specimens were deposited at the Herbarium of Hebei Normal University, China (HBNU), with specimen information provided in **Supplementary Table 1**, **Supplementary Figures 1**, **2**.

### 2.2 DNA extraction

Approximately 20 mg of dried leaf tissue was finely ground, and total genomic DNA was extracted from each sample using the Plant Genomic DNA Kit (DP305, TIANGEN BioTech (Beijing) Co., Ltd.) following the manufacturer’s standard protocol. The extracted DNA was used for subsequent experiments (**Supplementary Table 1**).

### 2.3 Development and validation of ITS molecular markers

In this study, the internal transcribed spacer (ITS) region was used as a representative nuclear gene fragment for the development and validation of molecular markers for 49 samples. The ITS fragments of all samples were amplified, with sequence information and amplification Tm values listed in **Supplementary Table 2**. PCR amplification products were sent to Sangon Biotech (Shanghai) Co., Ltd. for sanger sequencing.

The sanger sequencing data were corrected using Sequencher v5.4.5 (Gene Codes Corp., Ann Arbor, MI, U.S.A.). The corrected sequences were then automatically aligned with MAFFT v7.505, and the alignments were manually inspected and adjusted in BioEdit v7.0.8.0 to remove sequences with obvious alignment errors (Hall et al., 2011; Katoh et al., 2002). The alignments were then trimmed with TrimAl v1.4 to remove poorly aligned regions (Capella-Gutiérrez et al., 2009; Xiang et al., 2023). The resulting sequences were checked again and manually adjusted using BioEdit v7.0.8.0 (Hall et al., 2011). The ITS sequences of the *M. azedarach* ‘Mizhi’ were aligned with those of other cultivars to identify highly variable regions for primer design.

### 2.4 Acquisition of chloroplast whole genome

The chloroplast genomes of three *M. azedarach* cultivars, ‘Mizhi’, ‘Zijin’ and ‘Yuhua’, were obtained which were collected from Shijiazhuang Shenzhou Flower Institute Co. Ltd and voucher specimens were deposited at the Herbarium of Hebei Normal University, China (HBNU). Total DNA from the *M. azedarach* ‘Mizhi’, ‘Zijin’ (**Supplementary Table 1**) was fragmented into approximately 500 base pairs (bp) segments using a Covaris M220 ultrasonicator (Covaris, Woburn, MA, USA). DNA libraries were then constructed using the NEBNext Ultra II DNA Library Prep Kit, followed by low-coverage whole-genome sequencing on the BGI DNBSEQ-T7 platform (BGI, Shenzhen, China). The resulting paired-end raw sequencing data were subjected to quality control using FastQC v0.11.9 (Patel and Jain, 2012). Adapters and low-quality reads were removed with Fastp v0.23.4 (Chen et al., 2018), generating clean data. The clean reads were assembled using GetOrganelle v1.7.3.5. Based on sequence similarity with related species, preliminary annotation of the chloroplast genomes was performed using the online tools GeSeq (https://chlorobox.mpimp-golm.mpg.de/geseq.html) and CPGAVAS2 (http://47.96.249.172:16019/analyzer/home), and final manual annotation and correction were carried out using Geneious v9.0.2 (Jin et al., 2020; Kearse et al., 2012; Shi et al., 2019; Tillich et al., 2017). The chloroplast genome maps were drawn using the online tool OGDRAW (https://chlorobox.mpimp-golm.mpg.de/OGDraw.html) (Greiner et al., 2019). Protein-coding genes, tRNAs, and rRNAs in the chloroplast genomes were predicted using Geneious v9.0.2. Simple sequence repeats (SSRs) were identified and analyzed using the MISA website (http://pgrc.ipk-gatersleben.de/misa/). Using the MISA with default parameters, simple sequence repeats (SSRs) were defined as mono-, di-, tri-, tetra-, penta-, and hexanucleotide motifs with minimum repeat numbers of ten, six, five, five, five, and five, respectively. Compound SSRs were allowed with a maximum interruption distance of 100 bp between two adjacent SSRs. The IR and SC region boundary genes of the samples were analyzed using the IRscope tool (https://irscope.shinyapps.io/IRplus/) on the Bioinformatics Cloud platform (Amiryousefi et al., 2018). Comparative analyses were performed with chloroplast genomes of *M. azedarach* ‘Mizhi’, in addition, chloroplast genomes of Meliaceae downloaded from GenBank (**Supplementary Table 3**), together with *M. azedarach* ‘Mizhi’, were used to help determine the uniqueness of the cultivar-specific identification markers.

### 2.5 Development and validation of SNP molecular markers

The aligned chloroplast genome sequences of *M. azedarach* cultivars were analyzed, and nucleotide diversity (π) among the cultivars was calculated (using a window size of 600 bp and a step size of 200 bp) using DnaSP v6. Regions with relatively high π values were examined in detail to identify areas containing insertions/deletions or single-nucleotide polymorphism (SNP) markers specific to particular cultivars (Rozas et al., 2017). Six *M. azedarach* ‘Mizhi’ germplasm samples and six other germplasm samples (one from each, **Supplementary Table 1**) were used as experimental materials for PCR amplification and sequencing of the identified SNPs. Successfully amplified products were sent to Sangon Biotech (Shanghai) Co., Ltd. for sanger sequencing. Sequence analysis was performed adapted from the methods described in Section 2.3.

### 2.6 Microscopic and ultrastructural observation of leaf epidermal features

Dried leaf samples preserved with silica gel were used for observing epidermal structures for seven cultivars and 13 samples. For each sample, small leaf pieces approximately 1 × 0.5 cm were excised from both sides of the midrib and immersed in 6% sodium hypochlorite solution in a boiling water bath for 15 minutes, then cooled to room temperature. After the green color faded or the leaf edges turned whitish, the samples were removed and rinsed thoroughly with distilled water. The lower epidermis was peeled and mounted on temporary slides for observation under a light microscope. Images were captured at 400× magnification. For each sample, 30 representative fields between the leaf veins were examined, and mean values were calculated. The size and density of epidermal cells and stomata, as well as the stomatal index, were recorded (**Supplementary Table 1**).

Leaf epidermal ornamentation and stomatal features of five cultivars include 13 samples were examined using a low-vacuum scanning electron microscope (SEM, TM3030Plus, Hitachi). Before observation, each sample was cleaned with compressed air and then subjected to ultrasonic cleaning in anhydrous ethanol, followed by air-drying. Images were captured at magnifications of 200×, 500×, and 1000× using the SEM, and photographic records were taken (**Supplementary Table 1**).

### 2.7 Observation of leaf cross-sectional structure

Paraffin sections were prepared from the experimental materials through fixation, dehydration, wax infiltration, and embedding. After drying on a water bath and melting the paraffin, the sections were removed and stored at room temperature until use. The sections were stained with toluidine blue, mounted on slides, and examined under a light microscope. Cross-sectional structures of leaves from seven *M. azedarach* samples were observed, including the vascular tissues in the leaf veins as well as the palisade and spongy mesophyll tissues, and photographs were taken for documentation (Akinyele et al., 2021; Li and Bao, 2005).

## 3 Results

### 3.1 ITS sequence variation

**Figure 1:**
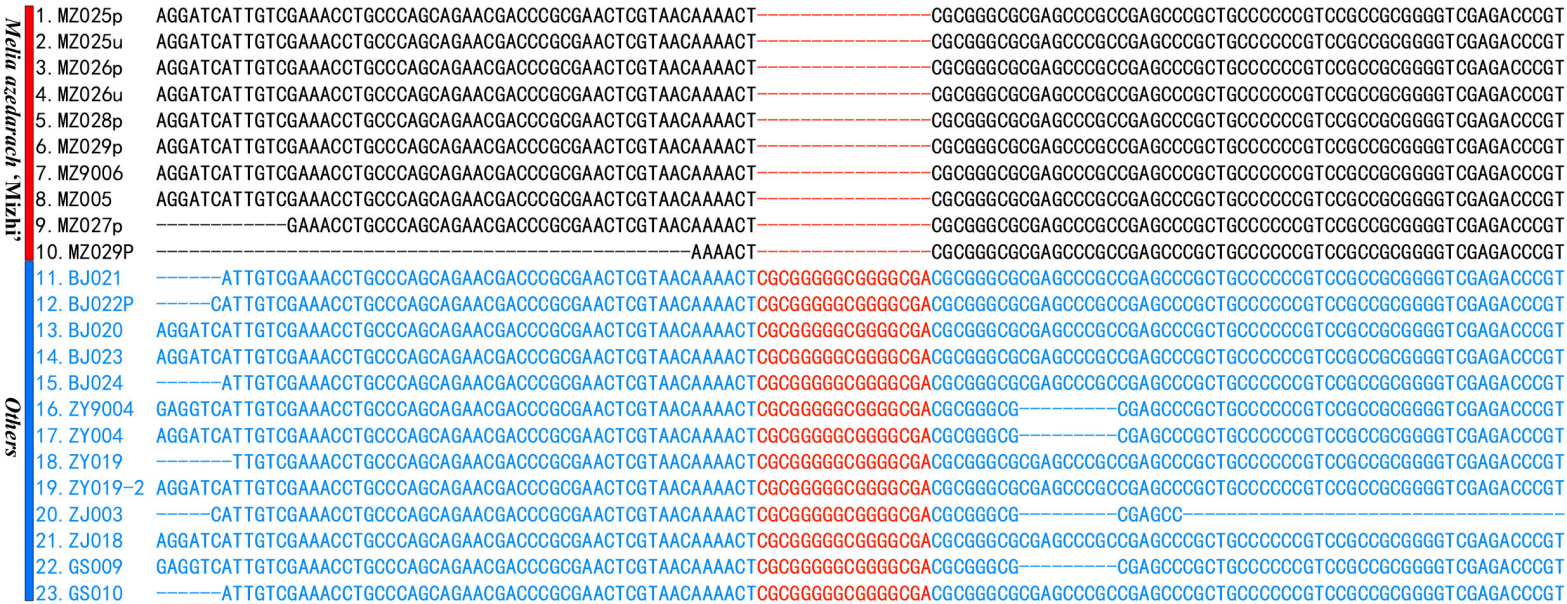
Alignment of ITS sequences among *M. azedarach* cultivars.

By comparing the ITS sequences of 49 samples, it can be observed that, all *M. azedarach* ‘Mizhi’ accessions showed a 16-bp deletion at positions 56-71 (**Figure 1**), whereas all other cultivars retained the sequence (5’-CGCGGGGGCGGGGCGA-3’). This deletion represents a stable and clearly identifiable marker for distinguishing *M. azedarach* ‘Mizhi’.

Primers were designed based on highly variable regions (5’-CGCGGGGGCGGGGCGA-3’) and PCR amplification was performed, followed by agarose gel electrophoresis (**Table 1**). The results showed that using the 16 bp sequence as a forward primer, PCR amplification was successful for both *M. azedarach* ‘Mizhi’ and other cultivars. This indicates that although Sanger sequencing revealed the 16 bp deletion in the ITS of *M. azedarach* ‘Mizhi’, the PCR amplification did not reflect the deletion’s effect (theoretically, no amplification product should have been obtained in *M. azedarach* ‘Mizhi’). Therefore, in practical applications, this deletion cannot serve as a definitive marker for identifying *M. azedarach* ‘Mizhi’ (**Supplementary Figure 3**).

**Table 1:** ITS primers for *Melia azedarach* ‘Mizhi’ identification.

Primer name	Sequence (5’-3’)	Tm value
Melia_Indel_F	ACTCGCGGGGGCGGGGCGA	73.11 °C
Melia_Indel_R1	CGATGCGTGACACCCAGGC	68.92 °C
Melia_Indel_R2	GACTGGAGCTCGAGAGGCTTG	62.44 °C
Melia_Indel_R3	CGTTCAAAGACTCGATGGTTC	57.18 °C

### 3.2 Chloroplast genome status

**Figure 2:**
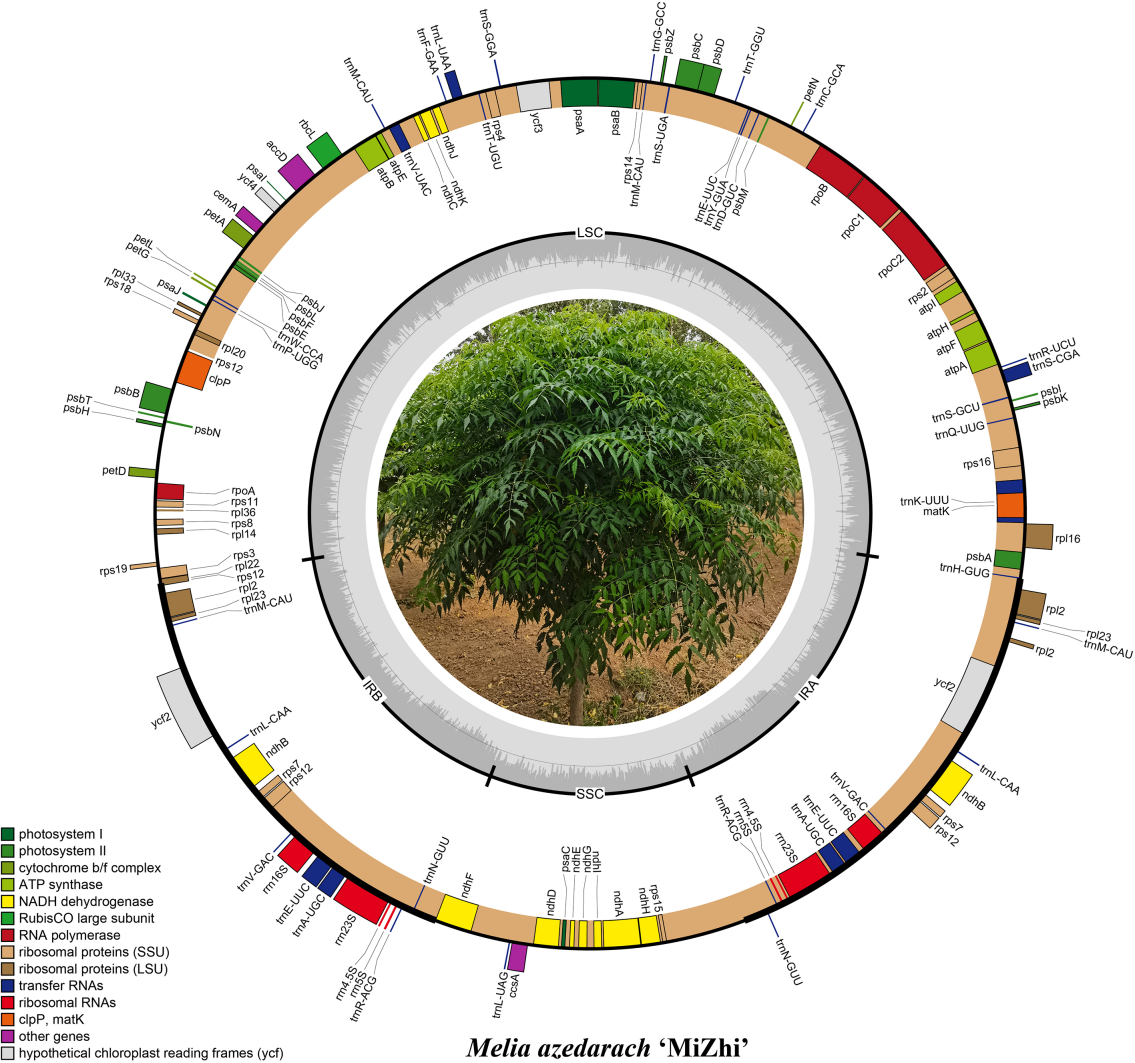
Chloroplast genome map of *M. azedarach* ‘Mizhi’. The inner circle depicts the GC content distribution across the genome.

**Figure 3:**
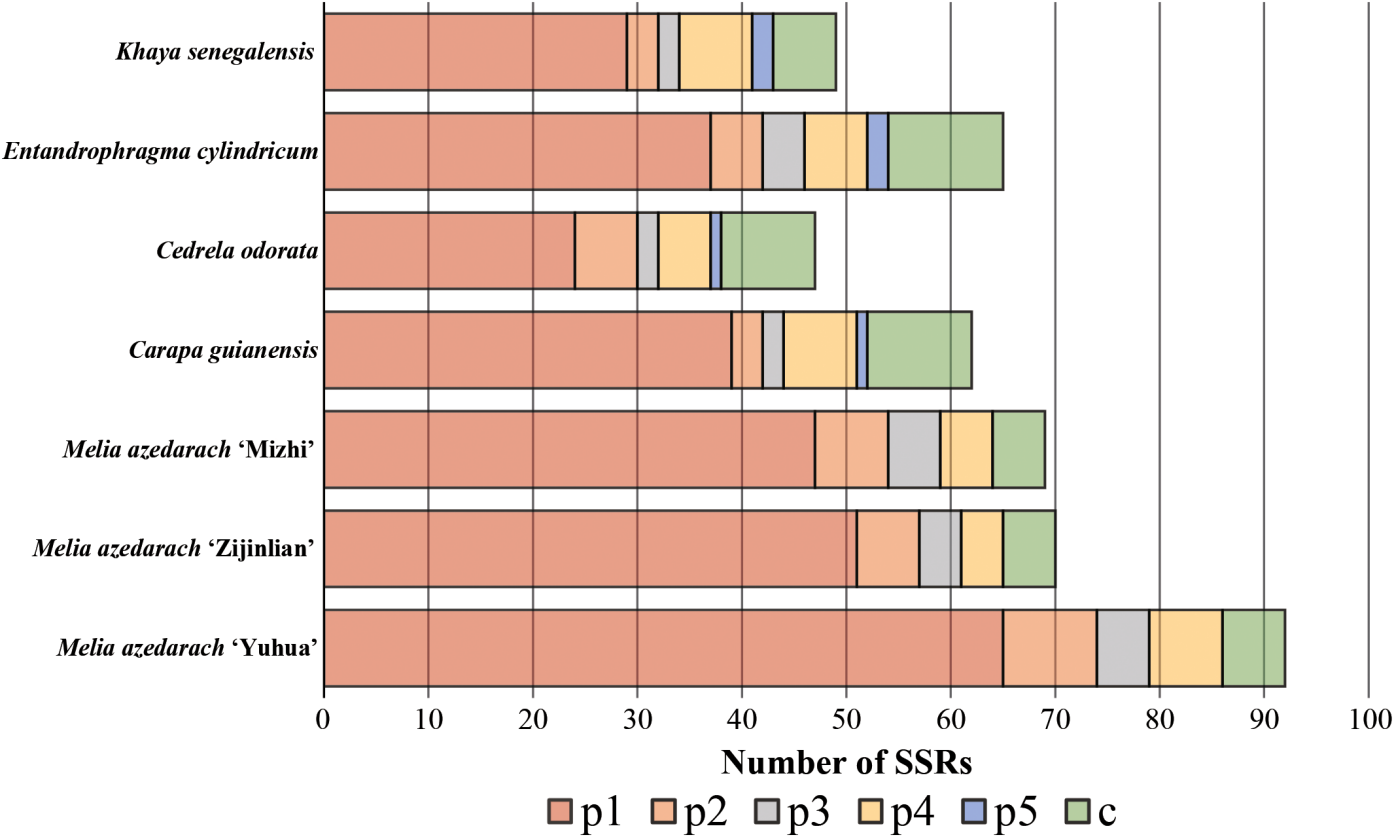
Distribution of simple sequence repeats (SSRs) in the chloroplast genomes of different *M. azedarach* cultivars and related species. p1-p5 represent mono-, di-, tri-, tetra-, and pentanucleotide repeat motifs, respectively, while C denotes compound SSRs.

The chloroplast genomes of the three *M. azedarach* cultivars exhibited a typical circular quadripartite structure, consisting of a large single-copy (LSC) region, a small single-copy (SSC) region, and two inverted repeat (IR) regions (**Figure 2**). The *M. azedarach* ‘Yuhua’ cultivar’s chloroplast genome was 161,805 bp in length, with an LSC of 92,287 bp, an SSC of 18,714 bp, and two IRs of 25,402 bp each. The total GC content was 37.3%. The genome encoded 127 genes, including 82 protein-coding genes, 37 tRNA genes, and eight rRNA genes. The *M. azedarach* ‘Zijin’ cultivar’s chloroplast genome was 161,805 bp long, with a GC content of 37.4%, comprising an LSC of 88,984 bp, an SSC of 18,741 bp, and two IRs of 26,519 bp each. This genome encoded 127 genes, including 82 protein-coding genes, 37 tRNA genes, and eight rRNA genes. The chloroplast genome of *M. azedarach* ‘Mizhi’ measured 162,341 bp, with a GC content of 37.3%, an LSC of 96,559 bp, an SSC of 18,744 bp, and two IRs of 23,519 bp each. It encoded 127 genes, including 82 protein-coding genes, 37 tRNA genes, and eight rRNA genes. Simple sequence repeats (SSRs) are important molecular markers that influence genome expansion and rearrangement. Across the seven samples, the number of SSR loci identified was 49, 65, 47, 62, 69, 70 and 92 in *Khaya senegalensis*, *Entandrophragma cylindricum*, *Cedrela odorata*, *Carpa guianensis*, *M. azedarach* ‘Mizhi’, *M. azedarach* ‘Zijin’ and *M. azedarach* ‘Yuhua’ respectively (**Figure 3**). Mononucleotide SSRs were dominant among all SSR types, and this pattern was consistent across all samples, followed by compound SSRs and dinucleotide SSRs. Among the samples, *Cedrela odorata* contained the fewest SSRs (47), whereas *M. azedarach* ‘Yuhua’ had the highest number (92). The total number of SSRs in *M. azedarach* ‘Mizhi’ was 69.

### 3.3 SNP status of cultivars of *M. azedarach*

Using DnaSP v6, regions with high nucleotide polymorphism (π) in the chloroplast genomes among the three cultivars were screened to identify areas containing insertions/deletions (Indels) or single-nucleotide polymorphisms (SNPs). These regions were then carefully manually verified. Primers were designed based on these regions, as listed in **Table 2**. PCR amplification using the SNP primers yielded DNA sequences of 126 bp. This region is located in the intergenic spacer (IGS) between the CDS *psbI* and *atpA*. Comparison of the SNP sequences across different germplasms revealed that *M. azedarach* ‘Mizhi’ differed from the others, exhibiting a single-nucleotide substitution at position 33, where C was replaced by A, while all other germplasms showed identical sequences. Therefore, the base at position 33 of the SNP sequence can be used as a reliable marker for the identification of the *M. azedarach* ‘Mizhi’ in germplasm authentication and conservation (**Table 3**).

**Table 2:** SNP primers for *Melia azedarach* ‘Mizhi’ identification.

Primer Name	Sequence(5’-3’)	PCR Program
SNP-F	GATTTGAACCTATACCAAAGG	94°C5min;[40cycles:94°C30s,50°C30s,72°C40s];72°C
SNP-R	GCATCGCTGAATTGAATACG	

**Table 3:** Specific SNP for *Melia azedarach* ‘Mizhi’ cultivars.

Name	Cultivar	Sequence (5’-3’)
SNP	*Melia azedarach*‘Mizhi’	ATTAGACATGGACGCCTTTC ATTCCGAATTTT**A**GCACTTT TATTTCCTGATCTCTTGTTC GGAAA
	Others	ATTAGACAATGGACGCCTT TCATTCCGAATTTTCGCAC TTTTATTTCCTATCTCTTGT TCGGAAA

### 3.4 Observation of leaf epidermal microscopic and ultrastructural features

#### 3.4.1 Leaf epidermal micromorphological characteristics

**Figure 4:**
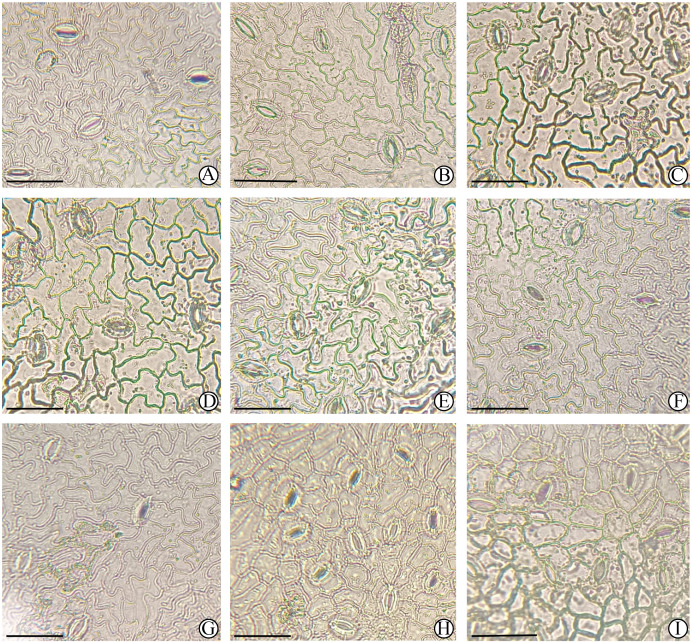
Leaf epidermal micromorphology of different *M. azedarach* cultivars observed under a light microscope, part I. All images show the abaxial epidermis. **(A)** *M. azedarach* ‘Mizhi’ 202209006, **(B)** *M. azedarach* ‘Mizhi’ WL005, **(C)** *M. azedarach* ‘Mizhi’ WL025, **(D)** *M. azedarach* ‘Mizhi’ WL026, **(E)** *M. azedarach* ‘Mizhi’ WL027, **(F)** *M. azedarach* ‘Mizhi’ WL028, **(G)** *M. azedarach* ‘Mizhi’ WL029, **(H)** *M. azedarach* ‘Beijing’ WL020, **(I)** *M. azedarach* ‘Yuhua’ WL017. Scale bar = 30 μm.

**Figure 5:**
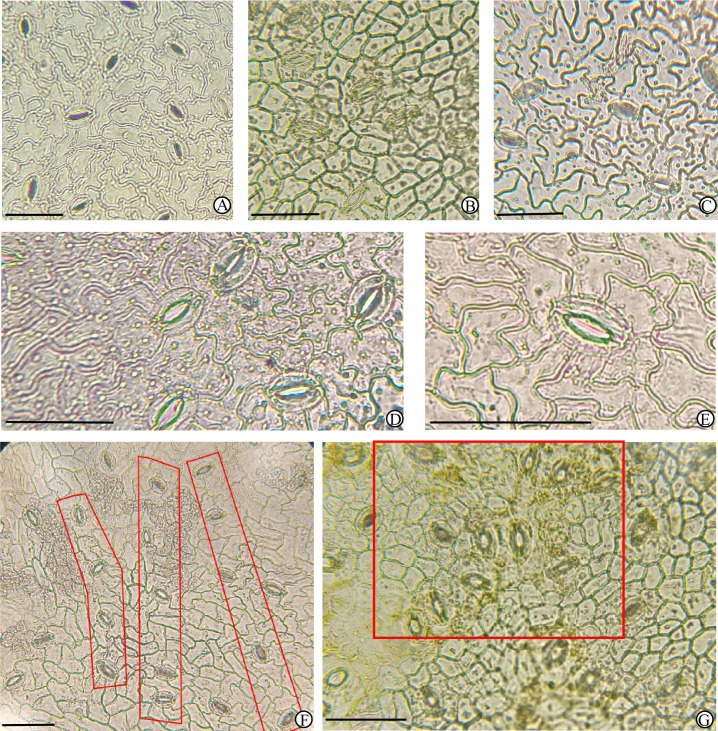
Leaf epidermal micromorphology of different *M. azedarach* cultivars observed under a light microscope, part II. All images show the abaxial epidermis. **(A)** *M. azedarach* ‘Gushu’ WL010, **(B)** *M. azedarach* ‘Nanling’ SZ6277, **(C)** *M. azedarach* ‘Zijin’ WL003, **(D)** *M. azedarach* ‘Ziyu’ WL004, **(E)** *M. azedarach* ‘Mizhi’ WL025, **(F)** *M. azedarach* ‘Mizhi’ showing banded distribution, **(G)** *M. azedarach* ‘Nanling’ showing clustered distribution. Scale bar = 30 μm.

As shown in **Table 4**, **Figures 4A–I**, and **Figures 5A–G**, stomata in the experimental samples were almost exclusively located on the abaxial (lower) leaf epidermis, with no stomata detected on the adaxial (upper) epidermis. The stomata are composed of two kidney-shaped guard cells, forming a relatively small elliptical opening (**Figure 5E**).

**Table 4:** Leaf epidermal micromorphological characteristics.

Collection number	Type	Stomatal size (µm)		Stomatal density(No./mm^2^)	Leaf epidermal cell morphology	Leaf epidermal cell area (um^2^)	Average number of leaf epidermal cells per field of view	Leaf epidermal cell density(No./mm^2^)	Stomatal index(%)
		Length	Width						
WL005	*Melia azedarach* ‘Mizhi’	14.42	7.68	314	Irregular-shaped	283.2	379.61	3217	9.74
WL025	*Melia azedarach* ‘Mizhi’								
WL026	*Melia azedarach* ‘Mizhi’								
WL027	*Melia azedarach* ‘Mizhi’								
WL028	*Melia azedarach* ‘Mizhi’								
WL029	*Melia azedarach* ‘Mizhi’								
202209006	*Melia azedarach* ‘Mizhi’								
WL020	*Melia azedarach* ‘Beijing’	14.53	6.84	655	Blocky polygonal	163.77	643.24	5451	12.07
WL017	*Melia azedarach* ‘Yuhua’	17.68	7.26	410	Blocky polygonal	187.83	579.86	4914	8.35
WL010	*Melia azedarach* ‘Gushu’	16.74	6.63	514	Irregular blocky	242.07	426.85	3617	14.83
SZ6277	*Melia azedarach* ‘Nanling’	17.89	8.63	268	Blocky polygonal	220.6	503.28	4265	6.97
WL003	*Melia azedarach* ‘Zijin’	13.37	8	302	Irregular-shaped	306.75	349	2958	10.31
WL004	*Melia azedarach* ‘Ziyu’	13.37	7.58	464	Irregular-shaped	253.04	411.56	3488	13.36

In the *M. azedarach* ‘Mizhi’ samples (202209006, WL005, WL025, WL026, WL027, WL028, and WL029), stomata were distributed relatively evenly and sparsely. The stomata were small, with a low stomatal density (314 counts/cells/units/mm^2^) and a low stomatal index (9.74%). Some stomata exhibited a banded distribution pattern (**Figure 5F**). Epidermal cells were relatively large (283.20 μm²), highly irregular in shape, and displayed pronounced undulating, wavy margins. The above characteristics are significantly different from those of other cultivars.

#### 3.4.2 Leaf Epidermis under scanning electron microscopy

**Figure 6:**
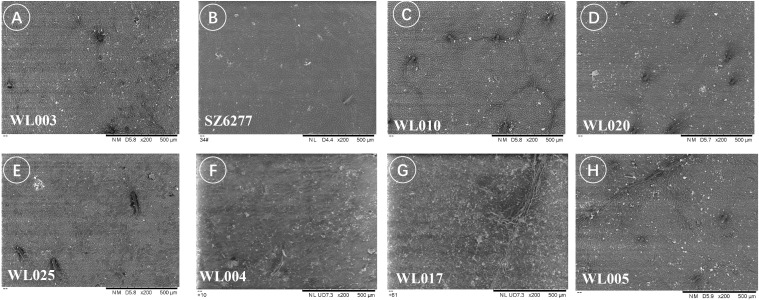
Variation in trichome morphology among different *M. azedarach* cultivars. Representative scanning electron micrographs of the adaxial (upper) leaf epidermis showing differences in trichome length and orientation. **(A)** WL003 (*M. azedarach* ‘Zijin’) with short, acicular, upright trichomes. **(B–H)** SZ6277 (*M. azedarach* ‘Nanling’), WL010 (*M. azedarach* ‘Gushu’), WL020 (*M. azedarach* ‘Beijing’), WL025 (*M. azedarach* ‘Mizhi’), WL004 (*M. azedarach* ‘Ziyu’), WL017 (*M. azedarach* ‘Yuhua’), and WL005 (*M. azedarach* ‘Mizhi’) with longer, acicular, curved trichomes.

Using scanning electron microscopy, trichomes were observed on the adaxial epidermis of all eight samples. Trichomes, as appendages of epidermal cells, were generally acicular in shape, but their characteristics varied among cultivars and could be used to identify some closely related cultivars (Cho et al., 2017; Wang et al., 2021). In *M. azedarach* ‘Mizhi’ (WL005), ‘Nanling’ (SZ6277), ‘Gushu’ (WL010), ‘Beijing’ (WL020), ‘Mizhi’ (WL025), ‘Ziyu’ (WL004), and ‘Yuhua’ (WL017), trichomes were longer, acicular, and curved (**Figures 6B–H**). In *M. azedarach* ‘Zijin’ (WL003), trichomes were short, acicular, and upright (**Figure 6A**).

**Figure 7:**
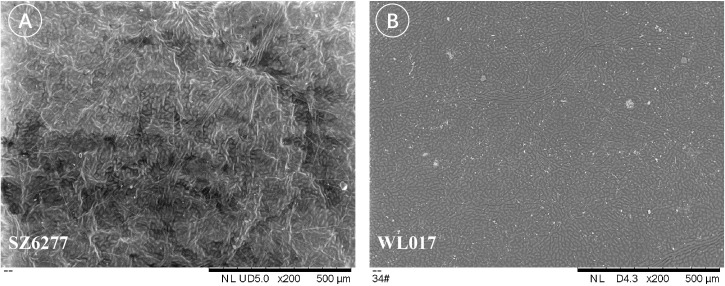
Regular epidermal cell morphology of the leaf cuticle in selected *M. azedarach* cultivars. Scanning electron micrographs showing adaxial epidermal cells with regular outlines. **(A)** SZ6277 (*M. azedarach* ‘Nanling’) displaying predominantly rectangular cells. **(B)** WL017 (*M. azedarach* ‘Yuhua’) showing polygonal epidermal cells with smooth margins.

**Figure 8:**
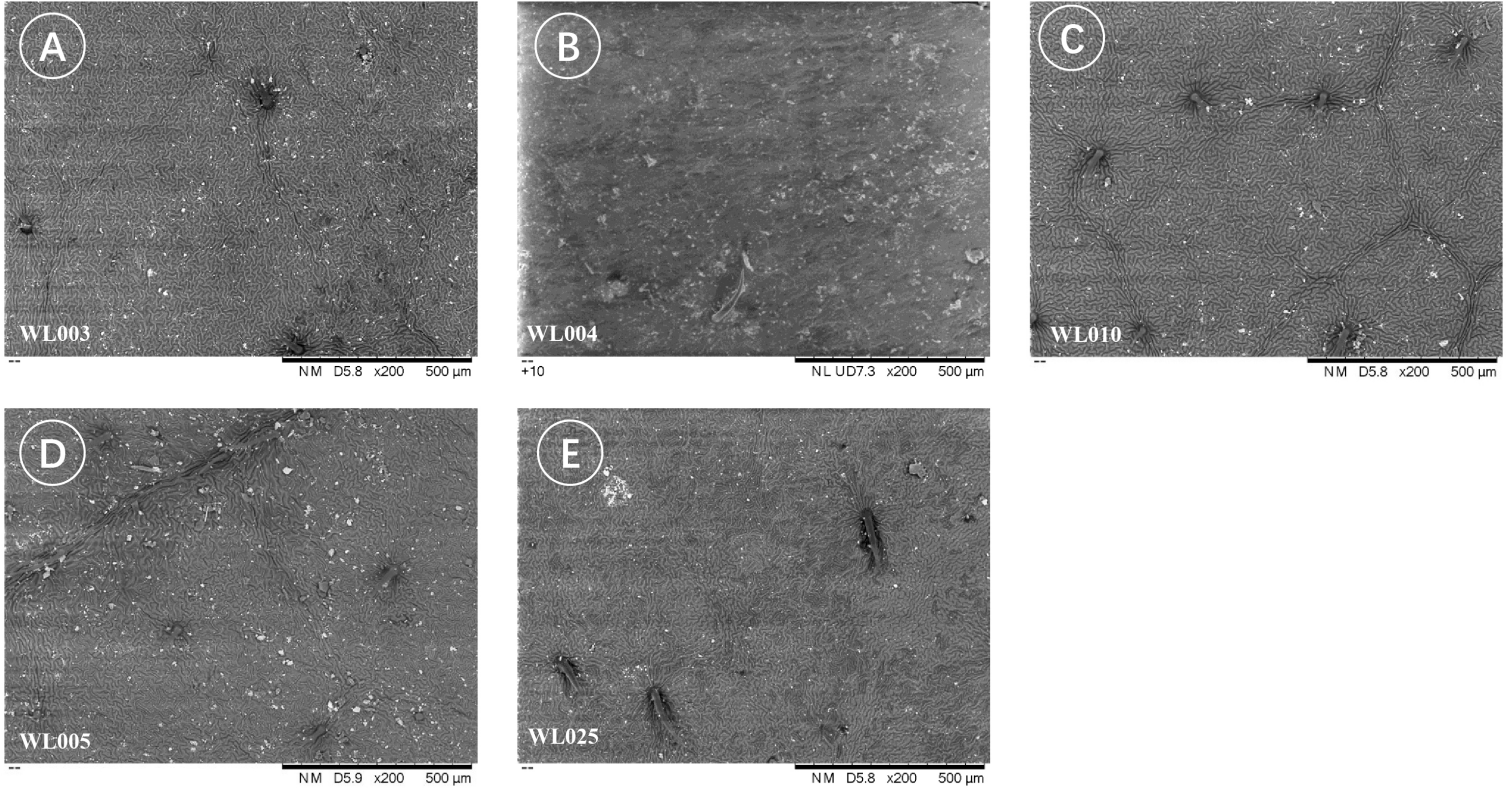
Irregular epidermal cell morphology of the leaf cuticle in selected *M. azedarach* cultivars. Scanning electron micrographs showing adaxial epidermal cells with wavy, irregular outlines. **(A)** WL003 (*M. azedarach* ‘Zijin’), **(B)** WL004 (*M. azedarach* ‘Ziyu’), **(C)** WL010 (*M. azedarach* ‘Gushu’), **(D)** WL005 (*M. azedarach* ‘Mizhi’), **(E)** WL025 (*M. azedarach* ‘Mizhi’).

Furthermore, differences in epidermal cell morphology of the leaf cuticle were observed, broadly categorized into two types. One type exhibited regular shapes, such as rectangular or polygonal cells (**Figures 7A, B**), as seen in *M. azedarach* ‘Nanling’ (SZ6277) and *M. azedarach* ‘Yuhua’ (WL017). The other type was irregular, with wavy cell walls (**Figures 8A–E**), observed in *M. azedarach* ‘Mizhi’ (WL005 and WL025), *M. azedarach* ‘Zijin’ (WL003), *M. azedarach* ‘Ziyu’ (WL004), and *M. azedarach* ‘Gushu’ (WL010).

Variations in stomatal density were also noted among cultivars. *M. azedarach* ‘Mizhi’ (WL005 & WL025) showed sparser stomatal distribution (**Figures 8D, E**), whereas *M. azedarach* ‘Gushu’ (WL010), *M. azedarach* ‘Yuhua’(WL017), and *M. azedarach* ‘Beijing’(WL020) exhibited relatively dense stomatal distribution (**Figures 8A–C**).

**Figure 9:**
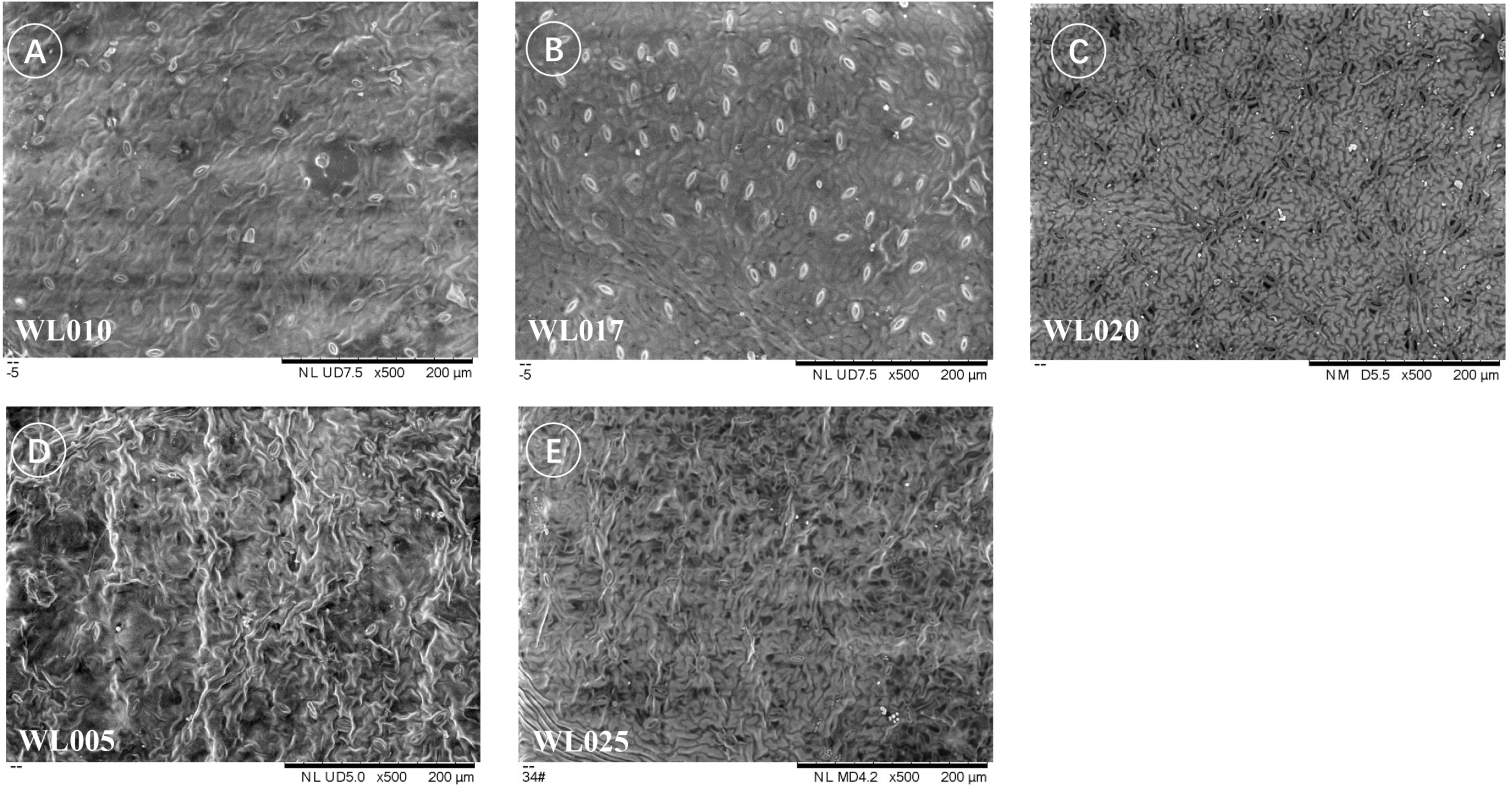
Variation in stomatal density among *M. azedarach* cultivars. Scanning electron micrographs of abaxial leaf surfaces illustrating differences in stomatal distribution. **(A)** WL010 (*M. azedarach* ‘Gushu’); **(B)** WL017 (*M. azedarach* ‘Yuhua’); **(C)** WL020 (*M. azedarach* ‘Beijing’); **(D)** WL005 (*M. azedarach* ‘Mizhi’); **(E)** WL025 (*M. azedarach* ‘Mizhi’).

Stomatal density also varied among the cultivars. *M. azedarach* ‘Mizhi’ (WL005 & WL025) showed comparatively sparse stomatal distribution (**Figure 9**), whereas *M. azedarach* ‘Yuhua’ (WL017), *M. azedarach* ‘Gushu’ (WL010), and *M. azedarach* ‘Beijing’ (WL020) exhibited relatively dense stomatal distribution.

### 3.5 Observation of leaf cross-sectional anatomy

**Figure 10:**
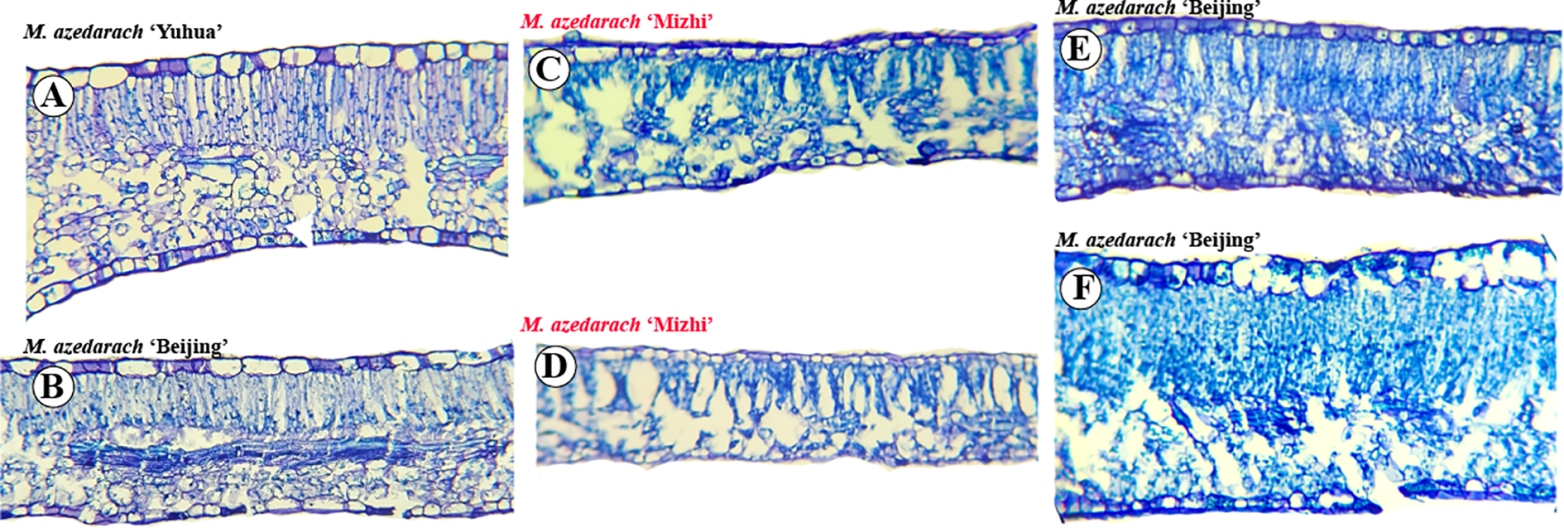
Vessel elements in leaf cross sections of M. azedarach cultivars. **(A)** *M. azedarach* ‘Yuhua’ (WL017), **(B)** *M. azedarach* ‘Beijing’ WL022, **(C)** *M. azedarach* ‘Mizhi’ WL025, **(D)** *M. azedarach* ‘Mizhi’ WL029, **(E)** *M. azedarach* ‘Beijing’ WL021, **(F)** *M. azedarach* ‘Beijing’ WL024. *M. azedarach* ‘Mizhi’ is highlighted in red font.

**Figure 11:**
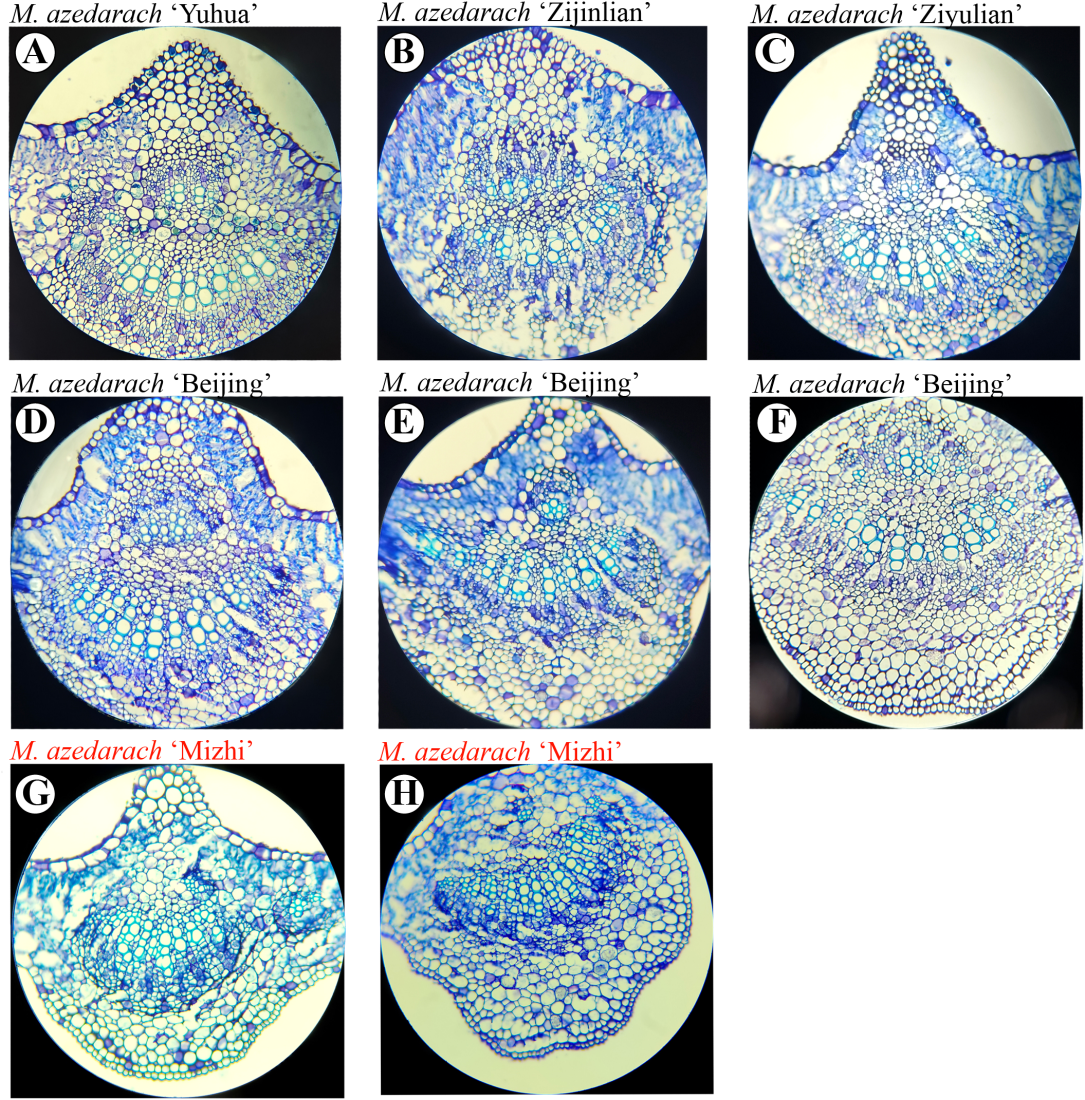
Lignified cell layers in leaf midrib cross sections of selected *M. azedarach* cultivars. Light micrographs illustrating differences in the number of lignified cell layers among cultivars. **(A)** *M. azedarach* ‘Yuhua’ (WL017), **(B)** *M. azedarach* ‘Zijin’ (WL018), **(C)** *M. azedarach* ‘Ziyu’ (WL019), **(D)** *M. azedarach* ‘Beijing’WL020, **(E)** *M. azedarach* ‘Beijing’ WL021, **(F)** *M. azedarach* ‘Beijing’ WL022, **(G)** *M. azedarach* ‘Mizhi’ WL025; **(H)** *M. azedarach* ‘Mizhi’ WL026.

The leaf structure was examined, revealing that both the adaxial and abaxial epidermis are covered by a cuticle. Epidermal cells on the adaxial surface are relatively large, whereas those on the abaxial surface are smaller, with abundant stomata. Beneath the adaxial epidermis, the palisade mesophyll is tightly arranged, while the spongy mesophyll near the abaxial epidermis is more loosely organized. *M. azedarach* ‘Mizhi’ leaves were thinner, with palisade and spongy tissues each occupying approximately half of the mesophyll, and the palisade arrangement appeared relatively loose in cross-section (**Figures 10**, **11**). Other cultivars had relatively thicker leaves.

## 4 Discussion

### 4.1 Morphological characters can assist in the identification of the *M. azedarach* ‘Mizhi’

The results of this study indicate that, according to the morphological descriptions of *M. azedarach* in the Flora of China, both the species and its intraspecific cultivars conform to the documented morphological traits. Therefore, conventional morphological characteristics cannot serve as distinguishing criteria between the *M. azedarach* ‘Mizhi’ and other cultivars. A comprehensive evaluation of leaf epidermal stomatal traits shows that the *M. azedarach* ‘Mizhi’ exhibits loosely and evenly distributed stomata, lower stomatal density and index, smaller stomata, and larger epidermal cells with irregular, wavy shapes. These features distinguish it from the *M. azedarach* ‘Beijing’, ‘Gushu’, ‘Zijin’, and ‘Ziyu’ cultivars (**Table 4**) (Sagaram et al., 2007, 2011; Zhang et al., 2012), differences between the *M. azedarach* ‘Mizhi’ and other cultivars or species can be observed through leaf cross-sectional structures. No unique forms specific to *M. azedarach* ‘Mizhi’ were detected by scanning electron microscopy; therefore, leaf epidermal micro-morphological traits and leaf cross-sectional structures can be used to distinguish ‘Mizhi’ germplasm. However, the observation and description of epidermal micro-morphology and leaf cross-sections require specialized expertise. If used as criteria for identifying ‘Mizhi,’ substantial knowledge in these areas is necessary. Furthermore, observations and descriptions of these traits may vary among different observers, and even a single observer may produce different descriptions at different times, introducing subjective bias. Thus, leaf epidermal micro-morphology and cross-sectional structures are not standardized markers and can only serve as supplementary indicators rather than definitive criteria for identifying the *M. azedarach* ‘Mizhi’ (Locatelli et al., 2019; Tao et al., 2018).

### 4.2 The internal transcribed spacer region holds potential feasibility for the identification of the *M. azedarach* ‘Mizhi’

Analysis of the ITS dataset at both the species and intraspecific levels of *Melia* revealed a 16 bp deletion uniquely present in the *M. azedarach* ‘Mizhi’, suggesting its potential utility as a diagnostic molecular marker for this cultivar (Álvarez and Wendel, 2003; Civetta, 2003). However, it is puzzling that PCR reactions using these 16-bp deletion sequence as a forward primer still produced ITS amplicons in the *M. azedarach* ‘Mizhi’. If the 16-bp sequence was truly deleted, a positive amplification would not be expected. This suggests that different ITS copies may exist within the ‘Mizhi’ genome, with at least one copy retaining the 16 bp sequence (Keirle et al., 2011; Zhang et al., 2022). Sanger sequencing determines the predominant base at each position based on fluorescent signal peaks (chromatogram peaks), effectively generating a consensus ITS sequence for a given species. Consequently, the 16-bp deletion may be overrepresented. Another possible explanation is that this region is relatively GC-rich. We also found that within the 16-bp deletion, six consecutive nucleotides are identical to those in the non-deleted sequence, which may promote nonspecific primer binding and subsequent off-target PCR amplification. Although *M. azedarach* ‘Mizhi’ cannot be directly identified based on agarose gel electrophoresis results, amplification of the entire ITS region followed by Sanger sequencing allows identification of *M. azedarach* ‘Mizhi’ by determining the presence or absence of the insertion. Therefore, ITS can serve as a molecular marker for identifying the *M. azedarach* ‘Mizhi’.

### 4.3 SNP markers hold great potential for the identification of *M. azedarach* cultivars

/The chloroplast whole-genome (CPG) data, from gene order and gene structure to overall variations, exhibit a high degree of conservation. Solely relying on chloroplast genome data as a super barcode cannot achieve specific identification of the *M. azedarach* ‘Mizhi’ (Li et al., 2015). Nevertheless, we identified a specific SNP locus for the *M. azedarach* ‘Mizhi’ in a localized region. To exclude potential random mutations introduced during the experiments, additional *M. azedarach* ‘Mizhi’ samples were validated using Sanger sequencing. The results confirmed a single-nucleotide substitution at position 33, where C is replaced by A, while sequences from other germplasms were identical. Therefore, for cultivar identification and protection, the nucleotide at position 33 of the SNP sequence can serve as a reliable marker for distinguishing the *M. azedarach* ‘Mizhi’, representing the optimal solution obtained in this study. Unlike morphological identification, whether considering the 16 bp deletion in ITS or the specific SNP variation, particularly the latter, the data are definitive, representing a clear presence or absence. Any investigator analyzing the same dataset will obtain consistent results, eliminating subjective bias. Moreover, specialized expertise is not required, as personnel following standardized procedures can achieve accurate identification. Considering the potential emergence of additional *M. azedarach* cultivars in the future, chloroplast genome and ITS data alone are unlikely to meet this need. However, the nuclear genome still contains abundant SNP loci, indicating that SNP-based markers hold great promise for cultivar identification in *M. azedarach*.

### 4.4 The importance of source data for the development of artificial intelligence in cultivar identification

With the rapid development and widespread adoption of artificial intelligence (AI), an increasing amount of data is being applied to cultivar identification. Under AI-driven frameworks, the accuracy of cultivar identification results depends critically on the quality of source data and the reliability of curated datasets. Therefore, beyond advances in algorithms and computational infrastructure, systematic exploration, curation, and integration of high-quality key data are essential. This, in turn, requires botanists to further identify, extract, and develop exploitable phenotypic traits and genetic information. Through such efforts can artificial intelligence fully realize its transformative potential in the field of cultivar identification, ultimately advancing cultivar identification, conservation, and utilization toward a new era that is more objective, efficient, and reliable.

## 5 Conclusion

This study analyzed three main types of *M. azedarach* cultivars (‘Yuhua’, ‘Zijin’, and ‘Mizhi’) from four perspectives: microscopic morphology, ultrastructure, nuclear gene fragments, and shallow genome sequencing. The results revealed that the *M. azedarach* ‘Mizhi’ exhibits loosely and evenly distributed stomata, low stomatal index and density, smaller stomatal size, larger epidermal cell area, and irregular, wavy epidermal cell shapes, distinguishing it from the *M. azedarach* cultivar ‘Beijing’, ‘Gushu’, ‘Zijin’, and ‘Ziyu’. Although these micromorphological traits can assist in identifying the *M. azedarach* ‘Mizhi’, the evaluation criteria are subjective, requiring substantial expertise and making standardization and practical application challenging. Chloroplast genome data, as a super barcode, cannot specifically identify the *M. azedarach* ‘Mizhi’. ITS sequences and SNP sequences show notable differences from other cultivars. SNP analysis revealed a unique variation in *M. azedarach* ‘Mizhi’ that can serve as a reliable and objective molecular marker, easily standardized for cultivar identification. This study provides new technical tools and theoretical support for the accurate identification and effective protection of the *M. azedarach* ‘Mizhi’ and offers important references for the sustainable development and utilization of *Melia* species, supporting the long-term conservation and rational utilization of valuable cultivar diversity. Finally, the standardized molecular markers and multi-scale phenotypic data generated in this study also provide high-quality source data for future AI-assisted and automated cultivar identification. This work lays a solid foundation for the development of objective, scalable, and data-driven identification systems in plant cultivar research.

## Data Availability

The CPG data obtained in this study have been deposited in Science Data Bank: https://doi.org/10.57760/sciencedb.34880.

## Supplementary material

Supplementary Figure 1 Herbarium specimens of the *M. azedarach* ‘Mizhi’. **(A)** WL028, **(B)** WL005, **(C)** WL026, **(D)** 202009006.

Supplementary Figure 2 Herbarium specimens of different *M. azedarach* cultivars. **(A)** WL028 (*M. azedarach* ‘Mizhi’) showing secondary rachis and leaflets, **(B)** WL021 (*M. azedarach* ‘Beijing’), **(C)** SZ6277 (*M. azedarach* ‘Nanling’), **(D)** WL019 (*M. azedarach* ‘Ziyu’) **(E)** WL018 (*M. azedarach* ‘Zijin’), **(F)** WL010 (*M. azedarach* ‘Gushu’), **(G)** WL017 (*M. azedarach* ‘Yuhua’).

Supplementary Figure 3 Agarose gel electrophoresis of PCR-amplified ITS hypervariable regions. **(A)** primer of *Melia*_Indel_F/*Melia*_Indel_R1; **(B)** primer of *Melia*_Indel_F/*Melia*_Indel_R2; **(C)** primer of *Melia*_Indel_F/*Melia*_Indel_R3 **(D)** primer of ITS U3/U4; M, maker; 1-47, the number of materials in **Supplementary Table S1**; 48, Negative control.
