# Supplementary figures and images for: Identification of intraspecific cultivar Melia azedarach ‘Mizhi’ based on complete chloroplast genome data and leaf anatomy
Source: Front Plant Sci. 2026 Mar 12;17:1783041. doi: 10.3389/fpls.2026.1783041 (PMC13018130; doi:10.3389/fpls.2026.1783041)

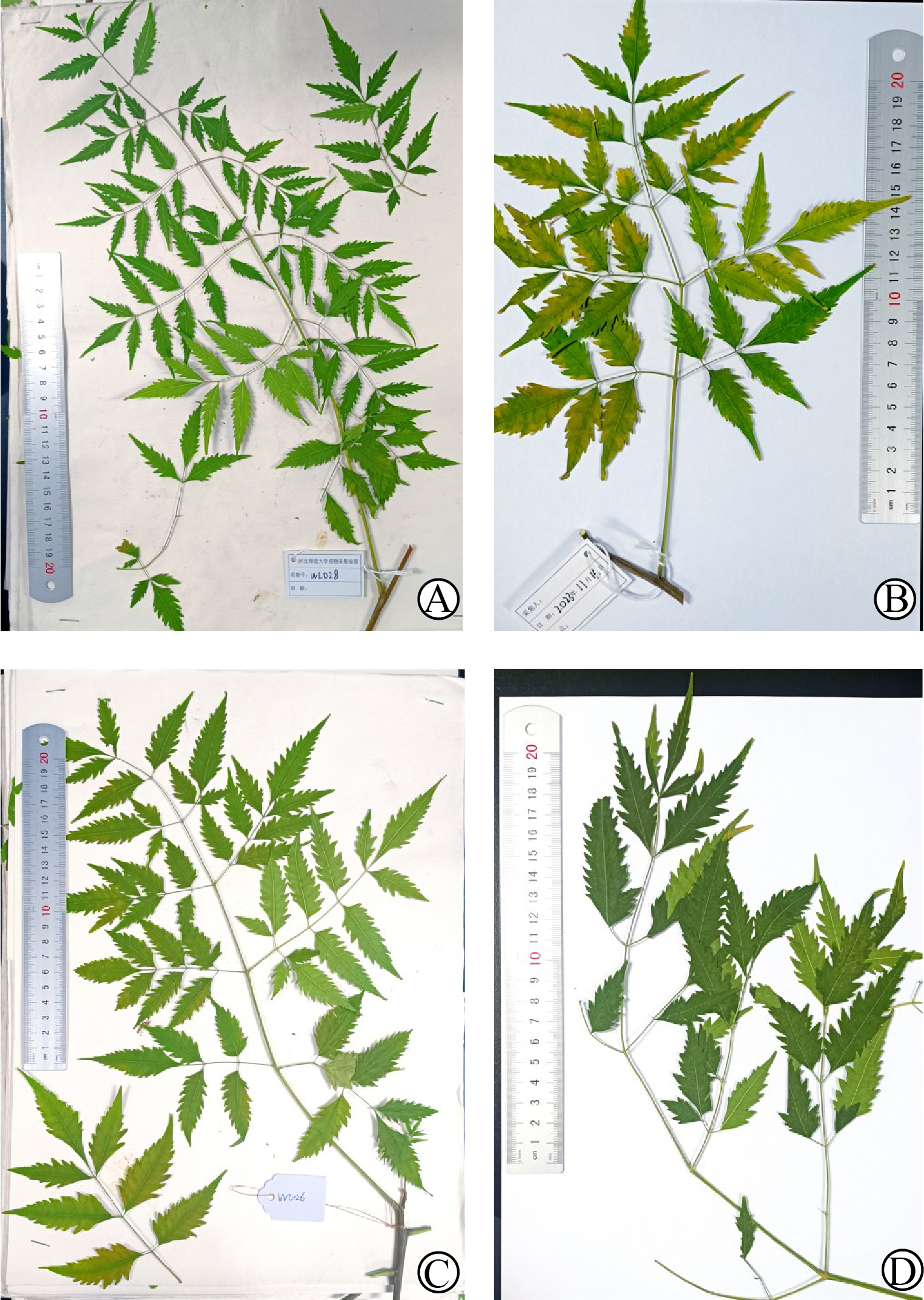

Supplement: Supplementary Figure 1 — Herbarium specimens of the M. azedarach ‘Mizhi’. (A) WL028, (B) WL005, (C) WL026, (D) 202009006. [file Image1.jpg]

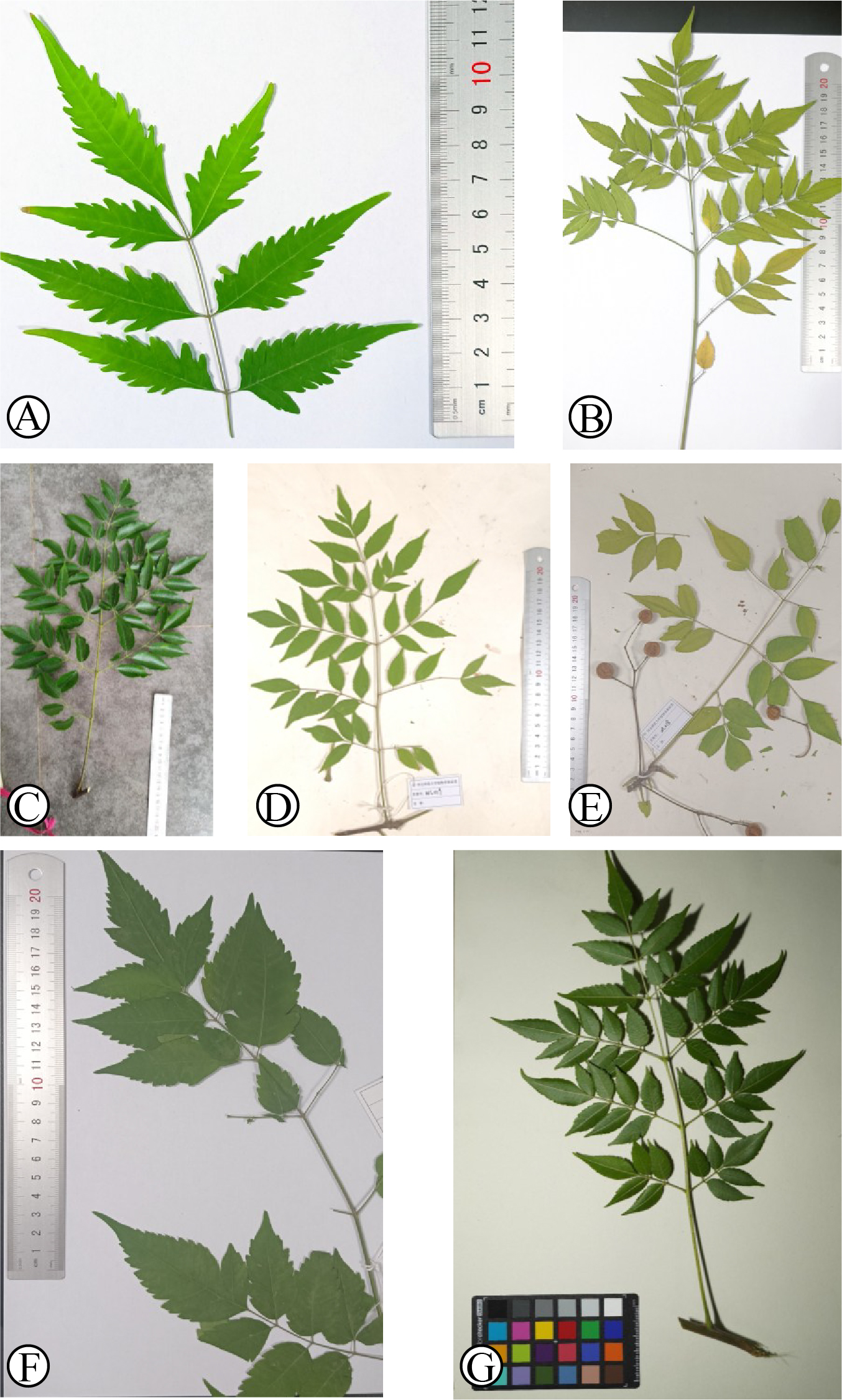

Supplement: Supplementary Figure 2 — Herbarium specimens of different M. azedarach cultivars. (A) WL028 (M. azedarach ‘Mizhi’) showing secondary rachis and leaflets, (B) WL021 (M. azedarach ‘Beijing’), (C) SZ6277 (M. azedarach ‘Nanling’), (D) WL019 (M. azedarach ‘Ziyu’) (E) WL018 (M. azedarach ‘Zijin’), (F) WL010 (M. azedarach ‘Gushu’), (G) WL017 (M. azedarach ‘Yuhua’). [file Image2.jpg]

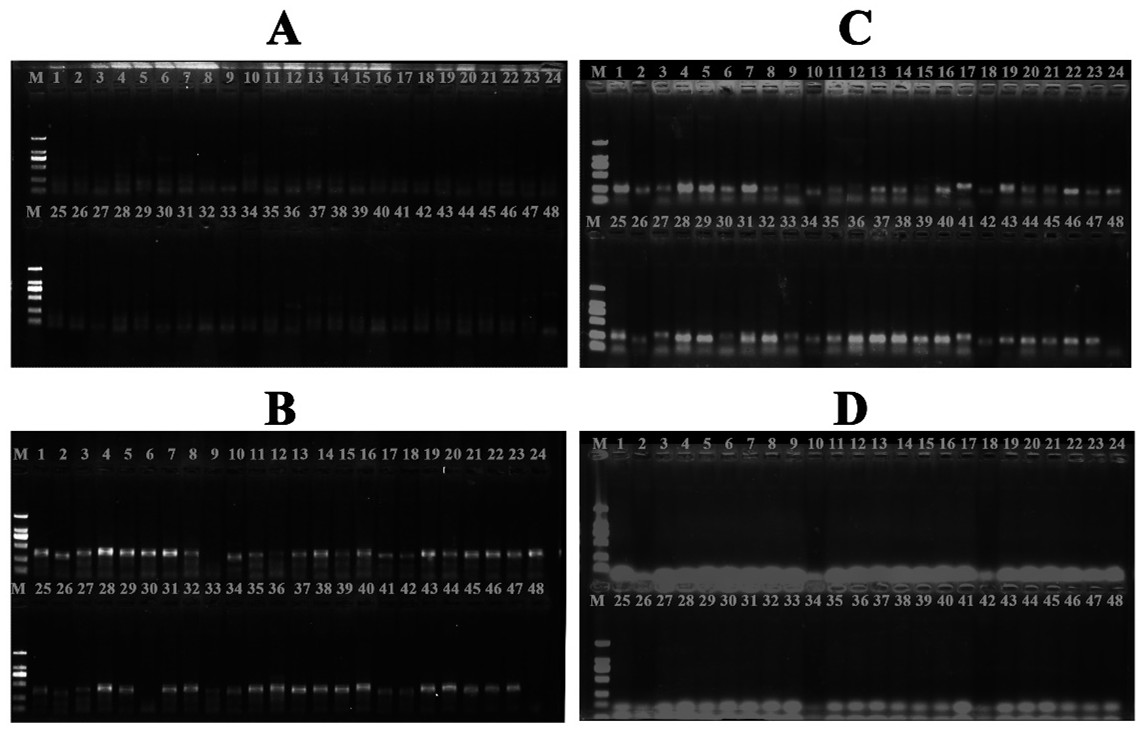

Supplement: Supplementary Figure 3 — Agarose gel electrophoresis of PCR-amplified ITS hypervariable regions. (A) primer of Melia_Indel_F/Melia_Indel_R1; (B) primer of Melia_Indel_F/Melia_Indel_R2; (C) primer of Melia_Indel_F/Melia_Indel_R3 (D) primer of ITS U3/U4; M, maker; 1-47, the number of materials in Supplementary Table S1; 48, Negative control. [file Image3.jpg]
