# Supplementary material for: Identification of intraspecific cultivar Melia azedarach ‘Mizhi’ based on complete chloroplast genome data and leaf anatomy
Source: Front Plant Sci. 2026 Mar 12;17:1783041. doi: 10.3389/fpls.2026.1783041 (PMC13018130; doi:10.3389/fpls.2026.1783041)
Supplement: Supplementary file 4 [file Table1.docx]

Table S1 Samples used in this study

| Source | Voucher | Leaf cross section | DNA barcoding | CPG genome | SNP and Indel | Observation of materials using a scanning electron microscope | Materials for comparing leaf epidermal micromorphological characteristics | Collect Area |
| --- | --- | --- | --- | --- | --- | --- | --- | --- |
| - | 202209001 |  | Yes | Yes |  |  |  | Yuhua, Shijiazhuang, Hebei Province |
| - | 202209002 |  | Yes |  |  |  |  | Yuhua, Shijiazhuang, Hebei Province |
| - | 202209003 |  | Yes |  |  |  |  | Yuhua, Shijiazhuang, Hebei Province |
| *Melia azedarach*‘Ziyu’ | 202209004 |  | Yes | Yes |  |  |  | Yuhua, Shijiazhuang, Hebei Province |
| - | 202209005 |  | Yes |  |  |  |  | Yuhua, Shijiazhuang, Hebei Province |
| *Melia azedarach*‘Mizhi’ | 202209006 |  | Yes | Yes |  |  | Yes | Yuhua, Shijiazhuang, Hebei Province |
| - | 202209007 |  | Yes |  |  |  |  | Yuhua, Shijiazhuang, Hebei Province |
| - | 202209008 |  | Yes |  |  |  |  | Yuhua, Shijiazhuang, Hebei Province |
| - | 202209009 |  | Yes |  |  |  |  | Yuhua, Shijiazhuang, Hebei Province |
| - | SZ6005 |  | Yes |  |  |  |  | Luquan, Shijiazhuang, Hebei Province |
| - | SZ6012 |  | Yes |  |  |  |  | Xingtang, Shijiazhuang, Hebei Province |
| - | SZ6013 |  | Yes |  |  |  |  | Xingtang, Shijiazhuang, Hebei Province |
| - | SZ6014 |  | Yes |  |  |  |  | Xingtang, Shijiazhuang, Hebei Province |
| - | SZ6015 |  | Yes |  |  |  |  | Xingtang, Shijiazhuang, Hebei Province |
| - | SZ6016 |  | Yes |  |  |  |  | Xingtang, Shijiazhuang, Hebei Province |
| - | SZ6017 |  | Yes |  |  |  |  | Xingtang, Shijiazhuang, Hebei Province |
| *Melia azedarach*‘Nanling’ | SZ6277 |  | Yes |  | Yes | Yes | Yes | Xinfeng, Ganzhou, Jiangxi Province |
| *Melia azedarach*‘Nanling’ | SZ6281 |  | Yes |  |  |  |  | Zhanggong, Ganzhou, Jiangxi Province |
| *Melia azedarach*‘Nanling’ | SZ6282 |  | Yes |  |  |  |  | Zhanggong, Ganzhou, Jiangxi Province |
| *Melia azedarach*‘Nanling’ | SZ6284 |  | Yes |  |  |  |  | Zhanggong, Ganzhou, Jiangxi Province |
| - | WL001 |  | Yes |  |  |  |  | Gaoyi, Shijiazhuang, Hebei Province |
| - | WL002 |  | Yes |  |  |  |  | Gaoyi, Shijiazhuang, Hebei Province |
| *Melia azedarach*‘Zijin’ | WL003 |  | Yes |  | Yes | Yes | Yes | Gaoyi, Shijiazhuang, Hebei Province |
| *Melia azedarach*‘Ziyu’ | WL004 |  | Yes |  |  | Yes | Yes | Gaoyi, Shijiazhuang, Hebei Province |
| *Melia azedarach*‘Mizhi’ | WL005 |  | Yes |  | Yes | Yes | Yes | Gaoyi, Shijiazhuang, Hebei Province |
| - | WL006 |  | Yes |  |  |  |  | Gaoyi, Shijiazhuang, Hebei Province |
| - | WL007 |  | Yes |  |  |  |  | Gaoyi, Shijiazhuang, Hebei Province |
| - | WL008 |  | Yes |  |  |  |  | Gaoyi, Shijiazhuang, Hebei Province |
| *Melia azedarach*‘Gushu’ | WL009 |  | Yes |  |  |  |  | Jingxing, Shijiazhuang, Hebei Province |
| *Melia azedarach*‘Gushu’ | WL010 |  | Yes |  | Yes | Yes | Yes | Jingxing, Shijiazhuang, Hebei Province |
| - | WL011 |  | Yes |  |  |  |  | Jingxing, Shijiazhuang, Hebei Province |
| - | WL012 |  | Yes |  |  |  |  | Jingxing, Shijiazhuang, Hebei Province |
| - | WL013 |  | Yes |  |  |  |  | Jingxing, Shijiazhuang, Hebei Province |
| - | WL014 |  | Yes |  |  |  |  | Jingxing, Shijiazhuang, Hebei Province |
| - | WL015 |  | Yes |  |  |  |  | Jingxing, Shijiazhuang, Hebei Province |
| - | WL016 |  | Yes |  |  |  |  | Yuhua, Shijiazhuang, Hebei Province |
| *Melia azedarach*‘Yuhua’ | WL017 | Yes | Yes |  | Yes | Yes | Yes | Yuhua, Shijiazhuang, Hebei Province |
| *Melia azedarach*‘Zijin’ | WL018 | Yes | Yes |  |  |  |  | Yuhua, Shijiazhuang, Hebei Province |
| *Melia azedarach*‘Ziyu’ | WL019 | Yes | Yes |  | Yes |  |  | Yuhua, Shijiazhuang, Hebei Province |
| *Melia azedarach*‘Beijing’ | WL020 | Yes | Yes |  | Yes | Yes | Yes | Yuhua, Shijiazhuang, Hebei Province |
| *Melia azedarach*‘Beijing’ | WL021 | Yes | Yes |  |  |  |  | Yuhua, Shijiazhuang, Hebei Province |
| *Melia azedarach*‘Beijing’ | WL022 | Yes | Yes |  |  |  |  | Yuhua, Shijiazhuang, Hebei Province |
| *Melia azedarach*‘Beijing’ | WL023 | Yes | Yes |  |  |  |  | Yuhua, Shijiazhuang, Hebei Province |
| *Melia azedarach*‘Beijing’ | WL024 | Yes | Yes |  |  |  |  | Yuhua, Shijiazhuang, Hebei Province |
| *Melia azedarach*‘Mizhi’ | WL025 | Yes | Yes |  | Yes | Yes | Yes | Yuhua, Shijiazhuang, Hebei Province |
| *Melia azedarach*‘Mizhi’ | WL026 | Yes | Yes |  | Yes |  | Yes | Yuhua, Shijiazhuang, Hebei Province |
| *Melia azedarach*‘Mizhi’ | WL027 | Yes | Yes |  | Yes |  | Yes | Yuhua, Shijiazhuang, Hebei Province |
| *Melia azedarach*‘Mizhi’ | WL028 | Yes | Yes |  | Yes |  | Yes | Yuhua, Shijiazhuang, Hebei Province |
| *Melia azedarach*‘Mizhi’ | WL029 | Yes | Yes |  | Yes |  | Yes | Yuhua, Shijiazhuang, Hebei Province |
