# Supplementary material for: Identification of intraspecific cultivar Melia azedarach ‘Mizhi’ based on complete chloroplast genome data and leaf anatomy
Source: Front Plant Sci. 2026 Mar 12;17:1783041. doi: 10.3389/fpls.2026.1783041 (PMC13018130; doi:10.3389/fpls.2026.1783041)
Supplement: Supplementary file 5 [file Table2.docx]

Table S2 ITS primers used in this study

| Fragment name | Primer Name | Sequence（5'-3'） | PCR Program |
| --- | --- | --- | --- |
| ITS | ITS-P5 | CCTTATCAYTTAGAGGAAGGAG | 94 ℃ 5min; [40 cycles：94 ℃ 30 s, 55 ℃ 30 s, 72 ℃ 40 s]; 72℃10min,16℃20min |
|  | ITS-U4 | RGTTTCTTTTCCTCCGCTTA |  |
