# Supplementary material for: Identification of intraspecific cultivar Melia azedarach ‘Mizhi’ based on complete chloroplast genome data and leaf anatomy
Source: Front Plant Sci. 2026 Mar 12;17:1783041. doi: 10.3389/fpls.2026.1783041 (PMC13018130; doi:10.3389/fpls.2026.1783041)
Supplement: Supplementary file 6 [file Table3.docx]

Table S3 Published chloroplast genome data of the Meliaceae

| Latin Name | Length (bp) | %GC | Accession Number |
| --- | --- | --- | --- |
| *Cedrela odorata* | 158,558 | 37.90% | NC037251 |
| *Entandrophragma cylindricum* | 159,609 | 37.80% | KY923074 |
| *Khaya senegalensis* | 159,787 | 37.90% | KX364458 |
| *Carapa guianensis* | 159,483 | 37.90% | MF401522 |
| *Melia azedarach* | 160,373 | 37.40% | PP099858 |
| *Azadirachta indica* | 160,737 | 37.50% | NC023792 |
